# Construction of A New Dose–Response Model for *Staphylococcus aureus* Considering Growth and Decay Kinetics on Skin

**DOI:** 10.3390/pathogens8040253

**Published:** 2019-11-21

**Authors:** Elaheh Esfahanian, Umesh Adhikari, Kirk Dolan, Jade Mitchell

**Affiliations:** 1 Department of Biosystems and Agricultural Engineering, Michigan State University, East Lansing, MI 48824, USA; esfahan3@msu.edu (E.E.); adhika12@msu.edu (U.A.); dolank@anr.msu.edu (K.D.); 2 Department of Food Science and Human Nutrition, Michigan State University, East Lansing, MI 48824, USA

**Keywords:** *S. aureus*, growth and decay, dose-response, Gompertz model, inverse problem

## Abstract

In order to determine the relationship between an exposure dose of *Staphylococcus aureus* (*S. aureus*) on the skin and the risk of infection, an understanding of the bacterial growth and decay kinetics is very important. Models are essential tools for understanding and predicting bacterial kinetics and are necessary to predict the dose of organisms post-exposure that results in a skin infection. One of the challenges in modeling bacterial kinetics is the estimation of model parameters, which can be addressed using an inverse problem approach. The objective of this study is to construct a microbial kinetic model of *S. aureus* on human skin and use the model to predict concentrations of *S. aureus* that result in human infection. In order to model the growth and decay of *S. aureus* on skin, a Gompertz inactivation model was coupled with a Gompertz growth model. A series of analyses, including ordinary least squares regression, scaled sensitivity coefficient analysis, residual analysis, and parameter correlation analysis were conducted to estimate the parameters and to describe the model uncertainty. Based on these analyses, the proposed model parameters were estimated with high accuracy. The model was then used to develop a new dose-response model for *S. aureus* using the exponential dose–response model. The new *S. aureus* model has an optimized k parameter equivalent to 8.05 × 10^−8^ with 95^th^ percentile confidence intervals between 6.46 × 10^−8^ and 1.00 × 10^−7^.

## 1. Introduction

*Staphylococcus aureus* is a common gram-positive bacterium of clinical significance causing skin and soft-tissue infections worldwide [1,2]. Approximately 10% to 30% of the population is estimated to be colonized with *S. aureus* on the skin or in the nose [3]. However, development of antibiotic-resistant strains such as methicillin-resistant *S. aureus* (MRSA) has become a major health concern, especially for hospital settings and community-acquired infections [4,5]. MRSA is often found at higher incidence in healthcare settings as compared to community settings. In a U.K. study, nearly 2% of patients were colonized after admission [6], while a U.S. study estimates that 4% of hospital inpatients are colonized [7,8]. Klevens et al. [9] reported about 9000 observed cases of MRSA per year in the U.S., in which 58.4% were associated with healthcare settings and 26.6% were community-based. The national burden of MRSA infections in the U.S. in 2014 was about 72,000 infections [10]. The Centers for Disease Control and Prevention Emerging Infections Program (EIP) population-based surveillance from 2009 to 2013 found a total of 4607 nursing-home onset and 4344 hospital-onset cases of invasive MRSA [11].

The anterior nares is the primary reservoir of the *S. aureus* in humans and the replication occurs followed by dispersal of the organism to the skin [12,13]. About 30% of all humans carry *S. aureus* in their nose persistently, while another 20% to 30% carry intermittently [14]. The typical transmission route of *S. aureus* is from the nose to the hand of a person [15], then to a surface (e.g., a door knob), and/or via the hand to the nose of a second person [16,17]. Activities involving close physical contact and the risk of minor injuries are positively correlated with *S. aureus* spread and acquisition [18]. Even a brief contact of fingers with a *S. aureus* contaminated surface may cause the transfer of a large amount of organisms resulting in a potential infection hazard [19]. The transfer rate is higher from moist contaminated surfaces than dry surfaces [20,21]. 

*S. aureus* can survive on dry surfaces between 2 and 4 days, and then can be easily transferred to hands and foods [22]. Other experiments showed more than a day of survival in hospital fabrics (cotton, terry, blend, and polyester) to over 90 days of survival in polyethylene [23]. These long survival times indicate a potential high risk of transmission of *S. aureus* through the surface-to-hand pathway. Once *S. aureus* is in the human body, it is believed to form biofilms, which makes the pathogen less vulnerable to host immune responses and allows them to cause colonization and local infections [14].

*S. aureus* is an opportunistic pathogen and does not usually pose a fatal risk to humans even if it colonizes human mucosa or skin [14]. However, in some cases, *S. aureus* can cause severe or fatal infections. *S. aureus* infections progress in five stages: colonization, local infection, systematic dissemination, metastatic infection, and toxinosis [24]. Severe forms of *S. aureus* infection include bacteremia, sepsis, pneumonia, endocarditis, and osteomyelitis [25]. The causative agent of 50% of all cutaneous infections is *S. aureus* [26,27]. Young children, the elderly population living in poor hygienic conditions, persons with diabetes and overweight conditions, and people living in high temperatures and humid conditions are particularly sensitive to *S. aureus* infection [28].

Quantitative microbial risk assessment (QMRA) is the process of characterizing health risk associated with pathogen exposures through environmental media [29]. QMRA follows a four-step paradigm similar to chemical risk assessment which begins with hazard identification, followed by an exposure assessment to quantify the number of organisms a receptor (i.e., human) comes in contact with based on the fate and transport of the organisms across an exposure pathway (i.e., hand-to-surface-to-mouth). Dose –response models are generally developed from controlled animal or human trials to describe the mathematical relationship between a given exposure dose and the probability of an adverse health outcome (i.e., infection, illness, or death). Such models are quasi-mechanistic in that they are derived from mathematical models that describe the plausibility of biological processes resulting in a measurable health endpoint [30] rather than the deep incorporation of mechanisms of in vivo physiological response. The final step in QMRA integrates the exposure dose prediction with the dose–response model to estimate risks with a characterization of the variability and uncertainty in the predicted values. 

For the majority of pathogens with peer-reviewed dose–response models (primarily for ingestion, inhalation, and similar exposure routes), no manipulation of the exposed dose in human and/or animal trials is required to fit a dose–response relationship. Due to the testing procedures used to estimate *S. aureus* infection—inoculation of the skin followed by occlusion which promotes growth—a transformation of the exposure dose is required prior to modeling the probability of infection [28]. This article describes the development of a *S. aureus* dose–response model using previously collected peer-reviewed data. The dose–response model is based on a model fit to a previously untested model to describe *S. aureus* growth on skin that captures the *S. aureus* growth and decay kinetics after inoculation (or exposure) with a low relative error and low correlation among estimated parameters as compared to the previous work in this area [28]. As *S. aureus* has recently risen to be among the leading causes of hospital-acquired infections, this new dose–response model should be a useful tool in estimating human *S. aureus* risk in order to support risk management evaluation (e.g., test behavioral changes on risk reduction or surface decontamination strategies). While the best fit parameter of the dose–response model remains unchanged over the previous work, uncertainty bounds around this estimate were desirable and the previous fit of the kinetic model could not be reproduced, thereby generating an opportunity to illustrate the inverse problem parameter estimation approach in a novel context.

## 2. Results and Discussion

### 2.1. Review of the Previous Model

**Figure 1:**
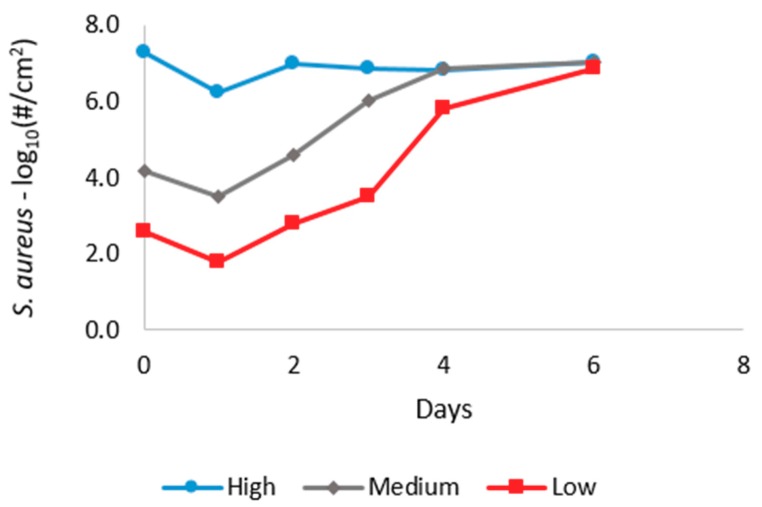
*S. aureus* growth and decay after inoculation.

**Figure 2:**
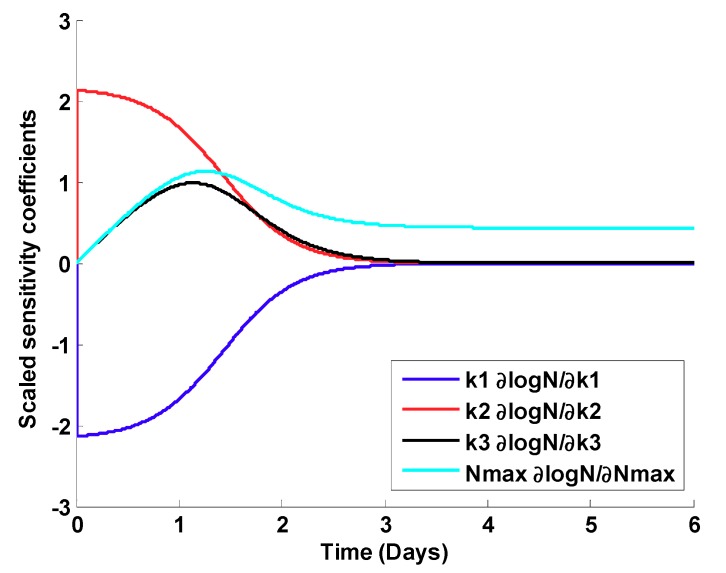
Scaled sensitivity coefficients of the parameter estimates from the first curve (high dose) for the model (Equation (1)) developed by Rose and Haas [28].

To evaluate the model developed by Rose and Haas [28], scaled sensitivity coefficients and correlation matrices for all the parameter estimates were created for each of the curves shown in Figure 1. Figure 2 shows the scaled sensitivity coefficients for the first curve (highest initial dose). As can be seen from the figure, the parameters are highly correlated, and after three days, it would be impossible to estimate most of the parameters. 

Table 1 shows the parameter correlation matrix of the parameter estimate corresponding to the first curve in Figure 1 (highest initial dose). As can be seen from the table, parameters *K_1_* and *K_2_* are highly correlated, making it hard to estimate the parameters separately.

### 2.2. Gompertz Growth and Decay Model

**Figure 3:**
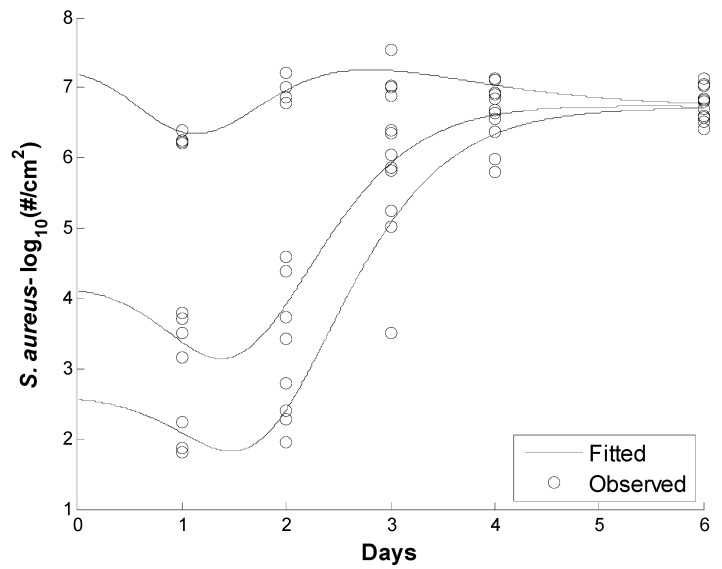
Data and fitted values from the Gompertz growth and decay model.

Various mathematical models has been used to capture the growth kinetics of *S. aureus* on different food products (i.e., cheese [31]; pork, ham, and sausages [32]; milk [33]; cooked potato and potato salad [34]; rice cake [35]; and sandwiches [36]). In these studies, a modified version of the following three models was used to model *S. aureus* growth: the Baranyi model [31,35,37]; the logistic model [32,33,34,38]; and the Gompertz model [36]. In this study, the Gompertz growth/inactivation models were used due to their capability to capture both the growth and decay kinetics of *S. aureus* on human skin (see Figure 3). Additionally, several studies have referred to the Gompertz model adaptively in capturing log-linear kinetics and shoulder and/or tailing effects, which is the case in Singh et al.’s data [39,40,41].

The results of parameter estimation to estimate *S. aureus* kinetic parameters are presented in this section. Figure 3 shows the observed and fitted values using the Gompertz growth and decay model. The figure shows that the model was able to capture the growth and decay kinetics of the *S. aureus* growth data.

Table 2 shows the mean parameter values, 95% confidence interval, and relative error of the estimated parameters. The results showed that the new model parameters could be estimated with higher confidence and low relative error.

**Figure 4:**
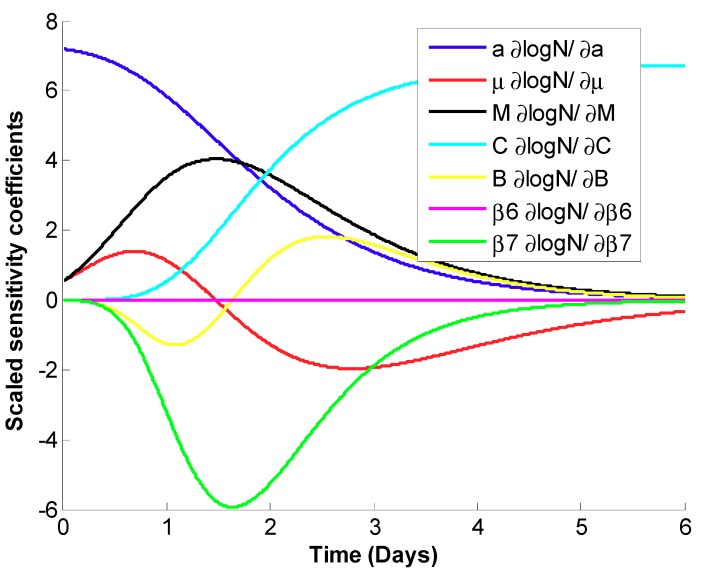
Scaled sensitivity coefficients of the parameters in the new *S. aureus* growth model (Equations (3) and (4)).

The scaled sensitivity coefficient plot shows that the parameters are independent of each other (Figure 4). Based on the absolute value of the scaled sensitivity coefficient plots, the parameters can then be estimated in the following order, from easiest to most difficult, *a*, *C*, *β7*, *M*, *μ*, *B*_,_ and *β_6_*, respectively. (The larger and more uncorrelated scaled sensitivity coefficients indicate computational ease in the estimation of the parameters.)

The correlation matrix of parameters is presented in Table 3. A smaller correlation between parameters indicates that parameters are more independent from each other and can be estimated better. The lowest correlation is found between *C* and *M*, and *B* and *β7* with the values of −0.26 and 0.04, respectively. Among all the parameters, the highest correlation is found between *M* and *β7* with a value of 0.85.

The estimated values of parameters obtained from OLS, the relative errors, and 95% confidence intervals for each parameter are given in Table 2. As predicted from Figure 3, the lowest relative error was for *C* and *β6*, which have the largest scaled sensitivity coefficients. All the parameters have a relative error below 11%. The root mean square error (RMSE) was 0.353, a low value compared to the total span of ~6 log (Figure 3).

### 2.3. Development of a New Dose–Response Model

Following the development of a new *S. aureus* growth model, the dose–response data presented in Table 4 were revisited and adjusted. Table 5 presents the revised dose–response data that account for the *S. aureus* growth and decay kinetics for the six days of occluding. 

**Figure 5:**
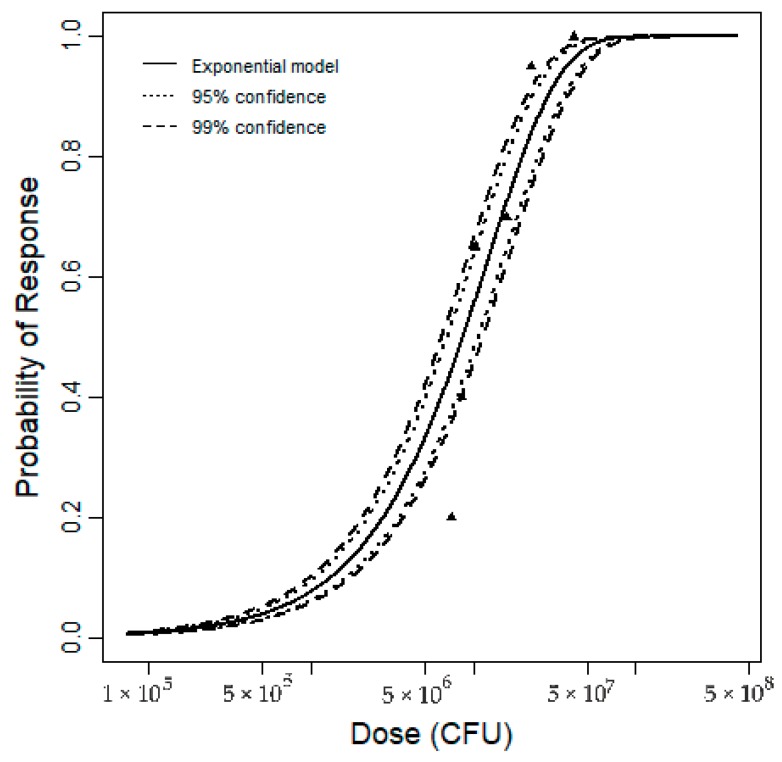
Best-fit dose–response model with 95% and 99% confidence intervals.

Revised doses from Table 4 were fitted into Equation (5) to obtain dose–response parameters. Figure 5 shows the revised doses, fitted model, observed and predicted risks along with the 95% and 99% confidence intervals.

Table 6 presents the *S. aureus* parameter values for the new dose–response model. The median value (or MLE) estimate was obtained by using the maximum likelihood estimation (MLE) method, and the uncertainty estimates are based on the bootstrapping resampling technique [29]. The best fit parameter, *k*, is equivalent to 8.05 × 10^−8^. The previously published model had a slightly higher *k* value, which would provide a more conservative estimate of risk.

## 3. Method

### 3.1. Development of the S. aureus Growth Model

#### 3.1.1. Data Source

In this study, data from Singh et al. [39] were used to develop *S. aureus* growth and decay models. Figure 1 shows the *S. aureus* growth data presented in Singh et al. [39] in which the growth of *S. aureus* on skin was investigated over 6 days after inoculation. The forearm skin of the human volunteers was initially inoculated with 7.30 [log_10_ (#/cm^2^)] (high), 4.18 [log_10_ (#/cm^2^)] (medium), and 2.60 [log_10_ (#/cm^2^)] (low) of *S. aureus* bacteria. The bacterial population kinetics of *S. aureus* were measured on 1, 2, 3, 4, and 6 days after application. As presented in Figure 1 in Section 2.1, each curve contains only six observations without any replication. Therefore, to fill the data gaps, three additional random data points were generated at each observation. The random points were generated using a normal distribution with a standard deviation of 0.3. 

#### 3.1.2. Review of the Previous Model

As a part of the model development process, the previous model developed by Rose and Haas [28] was analyzed. Equation (1) shows the *S. aureus* model developed by Rose and Haas [28]:

(1) $$\frac{dN}{dt}=-k_{1}Nexp\left(-k_{2}t\right)-k_{3}N\left(N_{max}-N\right)$$

where, *N* is the microorganism density (#/cm^2^), *N_max_* is the maximum microorganism density (#/cm^2^), *k_1_* is the initial inactivation rate constant (1/time), *k_2_* is the rate constant for the decrease in inactivation (1/time), and *k_3_* is the growth rate constant (cm^2^/#-time). The data were refitted to Equation (1) and the statistical parameters, correlation coefficient, and scaled sensitivity coefficient (see Section 2.1) were analyzed. Results for Equation (1) are shown in Figure 2 (Section 2.1).

#### 3.1.3. Gompertz Growth and Decay Models

In order to model *S. aureus* growth and decay on skin, the Gompertz inactivation model [42] was combined with the Gompertz growth model [43]. Each of these models has three parameters summing up to six parameters. The combined model is as follows:

*log N(t) = log N(0) {1-exp[-exp(-µ(t-M))]} + C exp{-exp[-B(t-L)]}* (2)

where *log N(t)* is the microbial concentration at time *t*, *log N(0)* is the initial microbial concentration (log_10_(#/cm^2^)), *µ* is the inactivation rate (day^-1^), *t* is the time (day), *M* is the lag factor (day), *C* is the difference between the upper and lower asymptote (log_10_(#/cm^2^)), *B* is the growth rate (day^-1^), and *L* is the time at which the inflection point occurs when the growth rate is maximum [41].

At the initial time (*t* = 0), Equation (2) does not equal *log N(0)* but rather equals to a multiple of *log N(0)*. Therefore, a new term, *a*, is introduced as the first parameter instead of *log N(0)*.

*log N(t) = a log N(0) {1-exp[-exp(-µ(t-M))]} + C exp{-exp[-B(t-L)]}* (3)

where, *a* is the ratio of *logN(t)* to *logN(0)* at *t* = 0.

Additionally, we noted that the parameter *L* in Equations (2) and (3) is linearly correlated to *log N(0)*. Therefore, we developed an empirical linear equation for *L* with two parameters, called *β_6_* and *β_7_*, as shown in Equation (4).

(4) $$L=\;\frac{\log N{\left(0\right)}_{max}-logN\left(0\right)}{\log N{\left(0\right)}_{max}-logN{\left(0\right)}_{min}}\;\beta_{6\;}+\;\frac{logN\left(0\right)-\log N{\left(0\right)}_{min}}{\log N{\left(0\right)}_{max}-logN{\left(0\right)}_{min}}\;\beta_{7\;}$$

where, *log N(0)_max_* is the maximum initial concentration in Singh et al.’s (1971) data (7.30 (log(#/cm^2^))), and *log N(0)_min_* is the minimum initial concentration in Singh data (2.60 (log(#/cm^2^))). Our hypothesis, based on the data, was that *L* linearly increased with log*N*(0).

#### 3.1.4. Parameter Estimation Methods

##### Ordinary Least Squares Estimation (OLS). 

The “nlinfit” command in MATLAB R2013b (Mathworks Inc., Natick, MA) was used to estimate the parameters by minimizing the sum of squares in the model, using the MATLAB nonlinear regression function, nlinfit. Detailed procedures to determine the confidence interval and the correlation matrix of parameters are given by Mishra et al. [44] and Dolan et al. [45].

##### Scaled Sensitivity Coefficients

Sensitivity coefficients are the first derivative of the model with respect to the parameter. Scaled sensitivity coefficients (SSCs) are the product of each parameter and its sensitivity coefficient, so that the SSC units are the same as those of the model. The SSCs visualize the sensitivity of the model to each parameter, and the dependency of parameters on each other in the model [41]. Larger and more uncorrelated scaled sensitivity coefficients indicate easier estimation of those parameters. A forward finite- difference method was used to compute the scaled sensitivity coefficients. 

### 3.2. Development of the S. aureus Dose-Response Model

#### 3.2.1. Data Source

Dose–response data were also obtained from Singh et al. [39]. The 20 test subjects were inoculated with six different doses of *S. aureus* and occluded for six days. Skin infection, which Singh et al. [39] described as “takes”, was appraised after six days. Table 4 shows the dose–response data after six days (see Section 2.3). 

#### 3.2.2. Revised *S. aureus* Dose

As Singh et al. [39] reported, there was *S. aureus* growth over the six-day period. Hence, the initial inoculation, as shown in Table 1, cannot be used as the dose. Therefore, Equation (3) was used to estimate the *S. aureus* growth over the six-day period. Initial doses presented in Table 4 were revised by calculating the area under the curve (AUC) for each day and summing up the dose for six days (see Table 5, Section 2.3).

#### 3.2.3. Fitting Dose–Response Model and Uncertainty Analysis

The revised data were used to fit the exponential dose–response model as shown in Equation (5).

(5) P(response)=1−exp(−k×dose) 

where *P(response)* is the risk of infection, *k* is the dose–response function parameter representing the probability that the organism survives to initiate infection (CFU^−1^), and *dose* is the exposure dose (CFU).

The model was fit in the R package (The R Project for Statistical Computing, r-project.org) using a maximum likelihood estimation (MLE). Confidence intervals were estimated using a bootstrapping technique, which provides a description of the uncertainty in the parameter estimated [29]. 

## 4. Conclusions

This study describes the development of a predictive microbial kinetic model to capture *S. aureus* growth and decay on human skin and a new fit of the *S. aureus* dose–response model which can be used to characterize the risk of infection through quantitative microbial risk assessment (QMRA) [29]. To our knowledge, it is the first study to use an inverse problem approach to estimate *S. aureus* kinetic parameters. The results indicate that the proposed model is highly capable of predicting *S. aureus* kinetics on human skin. The model parameters were easier to estimate and had lower relative error than those in Equation (1) (the Rose and Haas model [28]). This study also demonstrates that the inverse problem is a convenient approach in estimating the kinetic parameters of *S. aureus* on skin. The kinetic model developed would need to be modified in order to be used for other hosts (i.e., animals and food products). The newly developed kinetic model was also used to predict *S. aureus* growth in order to estimate the dose on the skin that produced observed infections in order to develop a new dose–response model. The new dose-response model and parameters can be useful to estimate the risk of human skin infection as the result of dermal contact with *S. aureus*. 

## Figures and Tables

**Table 1:** Correlation matrix of the parameter estimates from Rose and Haas [28].

	K_1_	K_2_	K_3_	N_max_
K_1_	1		Symmetric	
K_2_	0.9932	1		
K_3_	−0.0392	−0.0943	1	
N_max_	−0.2894	−0.2788	−0.7364	1

**Table 2:** Estimates of parameters with ordinary least square (OLS) and relative errors for the Gompertz model.

Parameters	Estimate	95% Confidence Interval		Relative Error (%)
*A*	1.00	0.94	1.05	2.63
*µ*	1.02	0.80	1.25	10.95
*M*	1.48	1.20	1.76	9.45
*C*	6.71	6.51	6.91	1.52
*B*	1.47	1.25	1.69	7.48
*β6*	2.35	2.25	2.45	2.1
*β7*	1.63	1.40	1.87	7.33

**Table 3:** Correlation matrix for the *S. aureus* growth model parameters.

	*A*	µ	M	C	B	β6	β7
a	1.00						
µ	−0.42	1.00			Symmetric		
M	−0.56	−0.07	1.00				
C	−0.08	0.37	−0.26	1.00			
B	−0.24	0.57	0.18	−0.28	1.00		
β6	−0.07	−0.37	0.51	−0.04	−0.13	1.00	
β7	−0.19	−0.34	0.85	−0.26	0.04	0.53	1.00

**Table 4:** *S. aureus* dose–response data.

Initial Dose (No./cm^2^)	Subjects with Infection	Total Subjects
40	4	20
220	8	20
2000	13	20
105,000	14	20
1,600,000	19	20
10,000,000	20	20

**Table 5:** Revised *S. aureus* dose–response data from Singh et al. [39].

Integrated Dose (AUC) (Days × No./cm^2^)	Subjects with Infection	Total Subjects
7.32 × 10^6^	4	20
8.45 × 10^6^	8	20
1.03 × 10^7^	13	20
1.59 × 10^7^	14	20
2.26 × 10^7^	19	20
4.15 × 10^7^	20	20

**Table 6:** Dose-response model parameters for *S. aureus.*

Parameter	MLE Estimate	Percentiles					
		0.5%	2.5%	5%	95%	97.5%	99.5%
k	8.05 × 10^−8^	6.06 × 10^−8^	6.46 × 10^−8^	6.70 × 10^−8^	9.69 × 10^−8^	1.00 × 10^−7^	1.08 × 10^−7^
